# Impact of a Resident-as-Teacher Point-of-Care Ultrasound (POCUS) Curriculum on Learner Confidence: A Prospective Educational Study

**DOI:** 10.7759/cureus.108218

**Published:** 2026-05-04

**Authors:** Guillermo Izquierdo Pretel, Emiri Uchiyama, Soheil Sadri, Mehak Sharma

**Affiliations:** 1 Internal Medicine, Jackson Memorial Hospital, Miami, USA; 2 Internal Medicine, Florida International University Herbert Wertheim College of Medicine, Miami, USA; 3 Medical Education, Florida International University, Miami, USA; 4 Internal Medicine, Florida International University, Miami, USA; 5 Internal Medicine, Bruce W. Carter Department of Veterans Affairs Medical Center, Miami, USA

**Keywords:** competency-based education, learner confidence, medical education, point-of-care ultrasound (pocus), resident-as-teacher, self-efficacy, simulation-based education, ultrasound training

## Abstract

Background: Point-of-care ultrasound (POCUS) is increasingly recognized as a core clinical skill in medical education. Resident-as-teacher models offer scalable educational approaches.

Objective: To evaluate the impact of a resident-led POCUS curriculum on learner confidence.

Methods: A prospective educational intervention was conducted at the Internal Medicine Department in Florida International University College of Medicine. Two identical five-hour workshops were delivered. Learners and instructors completed paired surveys. Data were analyzed using the Wilcoxon signed-rank test.

Results: Learners (n=9) demonstrated significant improvement in clinical application (p=0.031) and image interpretation (p=0.047), with mean confidence increasing across domains. Instructors (n=8) maintained high baseline confidence with no significant change.

Conclusion: A resident-led POCUS curriculum improves learner confidence and supports scalable education models.

## Introduction

Point-of-care ultrasound (POCUS) is increasingly recognized as an essential clinical skill in modern medical education; however, its implementation across training programs remains variable and often lacks standardization [1,2]. According to the American College of Physicians, only approximately 35% of Internal Medicine residency programs have a structured POCUS curriculum [3]. Confidence in clinical performance is closely related to self-efficacy, defined as the belief in one’s ability to successfully execute specific tasks [4]. A multi-site study has demonstrated that confidence in POCUS remains lower compared to other hospital medicine competencies [5]. Higher self-efficacy has been consistently associated with improved learning, skill acquisition, and performance among medical trainees [6]. Furthermore, confidence plays a critical role in competency development, facilitating progression along Miller’s pyramid from knowledge acquisition to clinical performance [4,7].

Structured, hands-on training has been shown to improve both learner confidence and competence in ultrasound-based skills [8,9]. In parallel, resident-as-teacher models have emerged as effective and scalable approaches to medical education, leveraging near-peer instruction to enhance learning outcomes while expanding teaching capacity [10,11].

Despite the growing recognition of POCUS as an essential clinical skill, there remains a need for effective and scalable educational strategies to enhance learner confidence and facilitate skill acquisition. In particular, the use of residents as instructors represents a promising but underexplored approach in POCUS education. Therefore, this study aimed to evaluate changes in learner confidence following a structured, resident-led POCUS workshop, using confidence as a proxy for self-efficacy and early competency development. We hypothesized that participation in this intervention would result in increased learner confidence, reflecting enhanced self-efficacy and early progression toward competency within established medical education frameworks.

## Materials and methods

Study design and setting

This prospective, single-arm educational intervention study was conducted at the Florida International University Herbert Wertheim College of Medicine (FIU HWCOM) Simulation Center. The study evaluated a resident-as-teacher POCUS workshop designed to improve learner confidence and early skill acquisition. The study was reviewed and deemed exempt by the FIU Institutional Review Board (IRB #IRB-25-0414).

Participants

Participants included medical students (MS3-MS4), postgraduate year (PGY)-1 internal medicine residents (learners), and PGY-2 and PGY-3 residents (instructors). Participants were recruited through institutional email invitations, academic schedules, and POCUS interest group announcements. Participation was voluntary, and informed consent was obtained prior to enrollment.

A total of approximately 50 individuals were invited to participate, of whom nine learners and eight instructors completed both pre- and post-workshop assessments and were included in the final analysis.

Intervention (POCUS workshop curriculum)

Two identical five-hour workshops were conducted. Each workshop consisted of a combination of brief didactic sessions and hands-on ultrasound training. The curriculum included established protocols such as extended Focused Assessment with Sonography in Trauma (eFAST), venous excess ultrasound (VExUS), and Rapid Ultrasound in Shock (RUSH), which are widely used for trauma evaluation, volume assessment, and hemodynamic assessment [12-14].

Participants rotated through multiple supervised scanning stations using simulation models and standardized participants. Each station focused on image acquisition, optimization, anatomical identification, and clinical interpretation. Resident instructors provided real-time feedback and facilitated skill development.

Data collection

Data were collected using structured pre- and post-workshop surveys administered via standardized forms. Surveys included demographic information (training level, prior ultrasound experience), self-reported confidence using a 5-point Likert scale (1=low confidence; 5=high confidence), and domains assessing image acquisition, interpretation, and clinical application (see Appendices).

Participants completed the pre-workshop survey prior to the session and the post-workshop survey immediately following completion of the workshop. All data were anonymized and stored on secure, password-protected institutional systems. No protected health information was collected.

Survey instrument

Pre- and post-workshop surveys were administered to both learners and instructors using a structured questionnaire. Confidence was assessed using a 5-point Likert scale (1=low confidence; 5=high confidence).

For learners, survey items evaluated confidence in multiple domains of POCUS competency, including identification of appropriate indications for POCUS (Q1), selection of ultrasound probe and machine settings (Q2), acquisition of high-quality ultrasound images (Q3), and interpretation of POCUS findings within a clinical context (Q4).

For instructors, pre- and post-workshop surveys assessed confidence in teaching and technical skills, including confidence in teaching POCUS (Q1), personal POCUS proficiency (Q2), image acquisition and quality optimization (Q3), and use of POCUS for clinical decision-making and teaching strategies (Q4).

In addition, instructors completed a post-workshop evaluation assessing perceptions of the teaching experience, including increased confidence as a clinical educator (Q1), effectiveness in teaching POCUS skills (Q2), adequacy of support and resources (Q3), and interest in participating in future teaching opportunities (Q4).

Outcome measures

The primary outcome was the change in learner confidence following the workshop. Confidence was evaluated across multiple domains of POCUS competency, including image acquisition, interpretation, and clinical application. Secondary observations included instructor confidence and qualitative feedback regarding the educational experience.

Statistical analysis

Descriptive statistics were used to summarize participant characteristics and confidence scores. Pre- and post-workshop confidence scores were compared using the Wilcoxon signed-rank test for paired data. Statistical significance was defined as a p-value <0.05.

## Results

A total of 17 participants completed both pre- and post-workshop assessments, including nine learners and eight instructors. Among learners, mean confidence scores increased across all assessed domains following the workshop. For Question 1 (image acquisition and interpretation), mean confidence increased from 2.23 pre-workshop to 3.42 post-workshop (p=0.071), demonstrating a trend toward improvement. For Question 4 (clinical application), mean confidence increased from 1.33 to 2.67, reaching statistical significance (p=0.031).

Among instructors, mean confidence scores also increased following the workshop. For teaching-related domains (Q1), mean confidence increased from 2.62 to 4.66, and for instructional strategy domains (Q4), from 2.50 to 5.00. Despite these observed increases, statistical comparisons did not reach significance (Q1: p=0.354; Q4: p=0.445).

Overall, the greatest improvements were observed among learners in domains related to clinical application of POCUS, while gains in image acquisition and interpretation demonstrated a positive but non-significant trend. Improvements were also observed among instructors, although these did not reach statistical significance. These findings highlight a consistent upward trend in confidence across both groups following the intervention.

**Figure 1:**
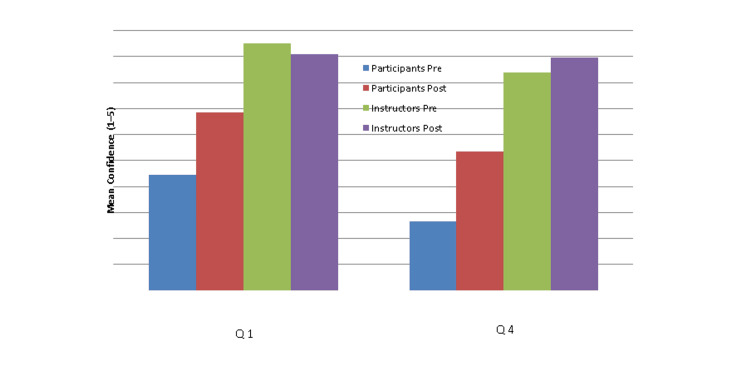
Pre- and post-workshop confidence scores among participants and instructors Participants demonstrated increased confidence following the workshop, particularly in clinical application, while instructors maintained high baseline confidence with minimal change, consistent with a ceiling effect. Q1: Image acquisition and interpretation; Q4: Clinical application and interpretation of findings.

Table 1 summarizes the pre- and post-workshop confidence scores for both participants and instructors, including mean values, standard deviations, and associated p-values. Figure 1 illustrates these changes graphically, demonstrating the increase in confidence across assessed domains following the intervention.

**Table 1:** Pre- and post-workshop confidence scores among participants and instructors Statistical comparisons were performed using the Wilcoxon signed-rank test. Values represent mean confidence scores ± standard deviation (SD) on a 5-point Likert scale (1=low confidence; 5=high confidence).

Group	Question	N	Pre (Mean±SD)	Post (Mean±SD)	p-value
Participants	Q1	9	2.89±0.93	3.89±0.60	0.071
Participants	Q4	9	2.89±0.78	3.67±0.71	0.031
Instructors	Q1	8	2.62±0.74	4.66±0.28	0.354
Instructors	Q4	8	2.50±0.53	5.00±0.00	0.445

## Discussion

Improvement in learner confidence likely reflects increased self-efficacy, a key determinant of skill acquisition [4,6]. It may reflect progression along the clinical competence continuum described by Miller, where increased confidence supports advancement from 'knows' and 'knows how' to 'shows how' in applied clinical skills [7]. Hands-on ultrasound training enhances both confidence and competence [8,15]. Resident-led teaching models are effective alternatives to traditional instruction [10,16]. The absence of change among instructors reflects a ceiling effect [17]. However, self-reported confidence may not fully correlate with objective competence [18].

Bandura’s theory of self-efficacy provides a useful framework to understand the impact of workshop-based learning. Self-efficacy, defined as an individual’s belief in their ability to successfully perform specific tasks, is influenced by four primary sources: mastery experiences, vicarious learning, verbal persuasion, and physiological or emotional states [4]. Hands-on workshops inherently integrate all four components. Participants engage in direct practice (mastery experiences), observe peers and instructors (vicarious experience), receive immediate feedback (verbal persuasion), and experience positive reinforcement upon achieving specific tasks (affective states). In the context of POCUS education, this includes successfully identifying anatomical structures such as the spleen, inferior vena cava, or performing a Doppler assessment of the portal vein. This comprehensive integration likely explains the observed improvements in learner confidence and supports workshops as an optimal modality for developing both self-efficacy and clinical competence.

In addition, confidence influences learner motivation and engagement. As described by Artino, learners with higher self-efficacy are more likely to select challenging tasks, persist in the face of difficulty, and maintain engagement in the learning process [6]. In the context of POCUS education, increased confidence following workshop participation may encourage learners to pursue further ultrasound training and apply these skills in clinical settings. This highlights the critical role of instructors in providing timely, constructive feedback to reinforce learner confidence and sustain motivation. By fostering a supportive learning environment, resident instructors can further enhance self-efficacy and promote continued skill development.

Furthermore, well-structured workshop-based activities align with key principles of effective simulation-based education. As highlighted by Issenberg et al., simulation is most effective when learners are provided with opportunities for deliberate practice, repetition, and immediate feedback [8]. Hands-on POCUS workshops inherently incorporate these elements, allowing learners to repeatedly perform tasks until a level of mastery is achieved. Rather than functioning as isolated educational events, such workshops can be integrated into a longitudinal curriculum to reinforce skill acquisition and retention. This approach supports the development of procedural competence and enhances the overall effectiveness of simulation-based medical education.

The role of self-assessment in medical education has been widely emphasized, particularly in the work of Eva and Regehr, who highlight its importance in promoting reflective learning and professional development [18]. In this study, confidence was measured using a semi-quantitative Likert scale, which represents a practical but indirect assessment of learner self-efficacy. While this approach provides valuable insight into perceived competence and learning progression, it may not fully capture actual performance or skill acquisition. This limitation underscores the complexity of measuring confidence and self-assessment in educational research. Nevertheless, such measures offer an important starting point for understanding learner development and provide a foundation for future studies incorporating more objective assessments.

Regardless of the clinical setting, the role of POCUS in modern medical practice is increasingly well established, supporting its integration into medical education through longitudinal curricula. As highlighted by Martin et al., POCUS training enhances anatomy understanding and physical examination skills in preclinical education, while also improving knowledge acquisition and procedural competency during clinical training across multiple specialties, including emergency medicine, surgery, and internal medicine [1]. Furthermore, continued integration into graduate medical education has been shown to improve both ultrasound knowledge and clinical competency. These findings reinforce the importance of incorporating structured POCUS training longitudinally across all stages of medical education, rather than as isolated learning experiences. In this context, workshop-based interventions may serve as foundational building blocks within a broader curriculum, facilitating progressive skill development and sustained competency.

Limitations

Limitations include the small sample size and reliance on self-reported confidence, which may not correlate with objective competence or clinical performance. Additionally, the study lacked objective measures of skill acquisition, such as standardized assessments or Objective Structured Clinical Examination (OSCE)-based evaluations. The single-institution design may also limit the generalizability of these findings.

## Conclusions

This study demonstrates that a resident-as-teacher POCUS curriculum is an effective and scalable educational strategy that enhances learner confidence, particularly in the clinical application of ultrasound. The observed improvements support the role of structured, hands-on workshops in fostering self-efficacy and facilitating progression along established competency frameworks in medical education. By integrating experiential learning, real-time feedback, and peer instruction, this model aligns with established theories of learning and highlights the value of resident educators in bridging gaps in ultrasound training.

These findings reinforce the importance of incorporating POCUS into longitudinal medical curricula and suggest that resident-led initiatives can play a critical role in expanding access to ultrasound education. Future studies should incorporate objective measures of competency and larger, multi-center cohorts to further validate these findings and strengthen the generalizability of this educational model.

## Appendices

Point-of-care ultrasound (POCUS) workshop questionnaires

A1. Learner Questionnaire

Pre-session survey

Participants were asked to complete the following survey prior to the POCUS workshop.

Role

☐ Medical Student

☐ Resident

☐ Physician

☐ Other

Prior POCUS Experience

☐ None

☐ Observed only

☐ Performed fewer than 10 examinations

☐ Performed more than 10 examinations

Confidence Assessment (5-point Likert scale: Not at all confident, Slightly confident, Moderately confident, Very confident, Extremely confident)

Please rate your confidence in the following areas:

1. Identifying appropriate clinical indications for POCUS

2. Selecting the correct transducer and machine settings for a POCUS examination

3. Acquiring diagnostically adequate POCUS images

4. Interpreting POCUS findings within a clinical context

Post-session Survey

Participants completed the following survey after the workshop.

Confidence Assessment (Post-Workshop) (5-point Likert scale as above)

Please rate your confidence in the following areas after completing the POCUS session:

1. Identifying appropriate clinical indications for POCUS

2. Selecting the correct transducer and machine settings for a POCUS examination

3. Acquiring diagnostically adequate POCUS images

4. Interpreting POCUS findings within a clinical context

Overall Satisfaction

How satisfied were you with the resident-led POCUS teaching session?

☐ Extremely satisfied

☐ Very satisfied

☐ Moderately satisfied

☐ Slightly satisfied

☐ Not satisfied at all

Perception of the Teaching Session (5-point Likert scale: Strongly disagree to Strongly agree)

Please indicate your level of agreement with the following statements:

1. The teaching session improved my understanding of ultrasound principles.

2. The resident instructors provided clear and helpful feedback during hands-on practice.

3. I feel more confident attempting POCUS independently after this session.

Open-Ended Questions

· What aspects of the session were most helpful?

· Suggestions for improvement:

A2. Instructor Questionnaire

Pre-session Instructor Survey

Postgraduate Year (PGY) Level

☐ PGY-1

☐ PGY-2

☐ PGY-3

☐ PGY-4 or higher

Prior Teaching or Ultrasound Training

Have you had formal training in teaching or ultrasound instruction?

☐ Yes

☐ No

If yes, please describe:

Confidence Assessment (5-point Likert scale: Not at all confident to Extremely confident)

Please rate your confidence in the following areas:

1. Teaching ultrasound skills to junior learners

2. Your own POCUS skills

3. Providing feedback on image acquisition and image quality

4. Familiarity with instructional strategies for ultrasound education

5. Using POCUS to guide clinical decision-making

Post-Session Instructor Survey

Teaching Experience Evaluation (5-point Likert scale: Strongly disagree to Strongly agree)

1. This teaching experience increased my confidence as a clinical educator.

2. I was able to effectively teach POCUS skills during the session.

3. I received adequate support and resources to deliver the session.

4. I am interested in participating in future POCUS teaching opportunities.

Open-Ended Questions

· What was most rewarding about this teaching experience?

· What challenges did you face while teaching?
